# A New Hospital System

**Published:** 1924-10

**Authors:** 


THE
### HOSPITAL and HEALTH REVIEW

### A NEW HOSPITAL SYSTEM.

There have of late been many significant signs that the voluntary hospitals of this country are approaching a new era in their history. One of the most unmistakable of these indications is furnished by the verbatim report of the recent Conferance between the Voluntary Hospitals Commission and the Local Voluntary Hospitals Committees which has just been issued. The perils into which the hospitals were plunged by the results of the Great War have produced, as escaped perils always should, a quickened consciousness. Both those who are immediately responsible and the external well-wishers and helpers who provide the means for the work that is being done recognise clearly that we have reached the parting of the ways; that if the voluntary system is to accomplish its destiny and cover ground at present vacant it must be carefully co-ordinated, more closely organised—become, in a word, a national system rather than a series of separate, and often competing, entities, however admirable those entities may be. All this was brought out with unusual completeness and vividness by the Conference, which discussed two subjects differing in kind rather than in importance—the inadequacy of the provision for patients, and the functions and future position of the Local Voluntary Hospitals Committees. Since, however, the former is the more pressing, it was naturally given the first place and received the fuller discussion. The investigations which the Committees have been making at the request of this Commission show not only that the additional number of beds required if the hospitals are to do their work thoroughly is very great, but that it is exceedingly difficult to arrive at how many really are needed. The great Royal Victoria Infirmary at Newcastle-on-Tyne alone asks for 800 or 900. Lord Cave declares roundly that even a few thousand more beds would be insufficient, and, looking at the problem as a national one, who shall say that his characteristically wide view is extravagant?

Before, however, we set to work to increase hospital accommodation on a large scale—which we shall unquestionably have to do before long—we have to assure ourselves that we are making the fullest use of what we already possess. It is certain that we are not, and that we never shall until our system is more comprehensive and resilient, and ceases to be divided into water-tight compartments. Our large general hospitals are cumbered with thousands of cases in various stages of recovery which ought to be drafted into institutions wholly or partially devoted to convalescents. The cottage hospitals are quite capable of dealing with the later stages of cases which it is not safe to send home because of the lack of facilities for treatment there With a network of regional federated hospitals the redistribution of beds would be a comparatively simple matter. Of far greater importance, however, is the waste of beds in the Poor Law Infirmaries. This great system of municipal hospitals functions side by side with the voluntary hospitals. In the best cases its institutions are as well equipped as most of the voluntary ones, and in almost every case they have during the last few years been enormously improved both in appliances and in the available medical and nursing skill. Yet while some voluntary hospitals are so crowded that emergency beds have to be provided on the spur of the moment, some of the great public hospitals under the care of the Guardians have a large proportion—sometimes as many as one half—of their beds unoccupied. This is not practical politics, but the difficulty is that occupancy of these municipal beds instantly converts the occupants into paupers. Lord Cave appears not to be quite sure whether the law really is what it has been judicially held to be, but it is time that any doubt there may be should be removed by a short Act of Parliament. That a man should be pauperised by a brief stay in a Poor Law Infirmary is a relic of the Dark Ages which ought to be swept away, even if there were no hospital question to give it urgency. Here, then, we have two means ready to hand by which the scarcity of beds in the voluntary hospitals might be substantially mitigated. These are not new discoveries, but they were discussed with great force and wealth of illustration at the Conference, and every day it becomes more important that action in these directions should be taken. Yet when we have derived the fullest advantage from these mitigations we shall be very far from having solved our difficulties. For the matter of that, we shall not know the extent of those difficulties until we have clear and definite information as to the effective numbers on the waiting lists. Some prospective patients place themselves on the lists of two or three hospitals to increase their chances, and there is one hospital which never revises its list. Moreover, a doctor often has a patient whom he would send to the hospital if it were possible. The Voluntary Hospitals Committees can do an essential piece of work by clearing away or greatly reducing the existing doubts about the extent of the real scarcity of beds.

To use these Committees for mere detail, however important, would, however, be to lose sight of their fundamental utility. Since their work in assisting the distribution of the Government Grant came to an end there has been a certain tendency to take it for granted that there is nothing left for them to do. But that was merely the end of their beginning—they are clearly marked out as the future co-ordinating machine of our hospital system and as a direct link between the hospitals and the Commission. They ought to be equally representative of the hospitals and the public who support them—that great public which at present has so little representation—and to have their own place on the Commission itself. To regard the Committees as mere *ad hoc* expedients would be to take the narrowest view of a subject which grows wider every year. With clear and definite objects they have before them a future of high importance. Exactly what those objects should be, the precise place which they should fill in the nation's hospital economy, has yet to be settled, and we are glad to learn that there is to be a further Conference for the discussion of this subject only. We can no longer afford to allow hospitals to work in an isolation which is not only wasteful in itself, but is full of danger to that voluntary principle which must be upheld with tireless tenacity.

